# ITD-YOLO: An Improved YOLO Model for Impurities in Premium Green Tea Detection

**DOI:** 10.3390/foods14091554

**Published:** 2025-04-28

**Authors:** Zezhong Ding, Yanfang Li, Bin Hu, Zhiwei Chen, Houzhen Jia, Yali Shi, Xingmin Zhang, Xuesong Zhu, Wenjie Feng, Chunwang Dong

**Affiliations:** 1 Tea Research Institute, Information and Economy Institution, Shandong Academy of Agricultural Sciences, Jinan 250100, China; dingzezhong1203@163.com (Z.D.); zv.chen@foxmail.com (Z.C.); 18006358878@163.com (H.J.); shiyali97@163.com (Y.S.); 18020970210@163.com (X.Z.); 15831111103@163.com (X.Z.); 2 College of Mechanical and Electronic Engineering, Shihezi University, Shihezi 832000, China; hb_mac@sina.com; 3 Weifang Engineering Vocational College, Weifang 262500, China; liyanfang3358@163.com

**Keywords:** tea, impurity identification, deep learning, Focaler_mpdiou, model pruning, knowledge distillation

## Abstract

During the harvesting and preparation of tea, it is common for tea to become mixed with some impurities. Eliminating these impurities is essential to improve the quality of famous green tea. At present, this sorting procedure heavily depends on manual efforts, which include high labor intensity, low sorting efficiency, and high sorting costs. In addition, the hardware performance is poor in actual production, and the model is not suitable for deployment. To solve this technical problem in the industry, this article proposes a lightweight algorithm for detecting and sorting impurities in premium green tea in order to improve sorting efficiency and reduce labor intensity. A custom dataset containing four categories of impurities was created. This dataset was employed to evaluate various YOLOv8 models, ultimately leading to the selection of YOLOv8n as the base model. Initially, four loss functions were compared in the experiment, and Focaler_mpdiou was chosen as the final loss function. Subsequently, this loss function was applied to other YOLOv8 models, leading to the selection of YOLOv8m-Focaler_mpdiou as the teacher model. The model was then pruned to achieve a lightweight model at the expense of detection accuracy. Finally, knowledge distillation was applied to enhance its detection performance. Compared to the base model, it showed advancements in P, R, mAP, and FPS by margins of 0.0051, 0.0120, and 0.0094 and an increase of 72.2 FPS, respectively. Simultaneously, it achieved a reduction in computational complexity with GFLOPs decreasing by 2.3 and parameters shrinking by 860350 B. Afterwards, we further demonstrated the model’s generalization ability in black tea samples. This research contributes to the technological foundation for sophisticated impurity classification in tea.

## 1. Introduction

Green tea is a distinctive category of tea in China [1]. Its production process generally includes picking, withering, greening, kneading, drying, and refining [2]. In the stages leading up to harvesting and throughout the processing of tea, it is inevitable that some impurities, which affect the quality of the tea, may be mixed in. Consequently, the sorting of these impurities is essential [3]. In light of acute labor shortages and the rapid advancement of automation technologies, there is a growing demand for the automation of sorting out impurities from tea, highlighting its escalating necessity. Currently, color sorting machines are pivotal in automating the process of eliminating tea impurities, leading to a substantial enhancement in sorting efficacy [4]. Nevertheless, for the impurities in famous green tea with a unique or fragile shape, the sorting ability of color sorters is still insufficient, and there are some potential disadvantages. One is that the tea will suffer friction-induced damage and breakage under mechanical stress, thereby compromising both its aesthetic appeal and overall structural soundness [5]. Second, when sorting some impurities similar to the shape of tea, a large number of qualified tea leaves will also be separated and removed together with the impurities, which affects the output of famous green tea. Third, the rapid sorting action performed by color sorters can exert an influence on the volatile compounds responsible for tea’s aroma profile, subsequently impacting its overall flavor characteristics [6]. Typically, color sorting technology is employed for filtering out imperfections in lower-grade tea, whereas the purification of superior green tea varieties continues to depend on manual methods, proving to be an expensive and physically demanding process [7]. Guo et al. introduced an approach for detecting impurities in Pu’er tea utilizing spectral imaging techniques. Hyperspectral imaging was employed to capture detailed images of the tea samples along with their associated impurities across a spectrum ranging from 400 to 1000 nanometers. From these images, spectral information was then gathered for every distinct type of sample, facilitating the creation of a Support Vector Machine (SVM) model, which enabled classification at the pixel level of the hyperspectral images [8]. While this technique proves efficient in pinpointing contaminants within Pu’er tea, its implementation in practical manufacturing settings remains limited due to the prohibitive expense associated with spectrometry equipment and the susceptibility of spectral reflection data to disruptions.

Recent years have witnessed remarkable progress in deep learning technology [9], particularly in the area of target detection algorithms. Such algorithms can be categorized into two main types, namely, a two-stage detection network, exemplified by Faster R-CNN, which has high detection accuracy and slow speed [10], and a single-stage detection network, such as SSD and YOLO [11], which has high detection speed but lower accuracy. In addition, the complexity of existing target detection models is high, which is not favorable for deployment in real production [12]. Therefore, it is essential to develop a high-precision and lightweight impurity detection algorithm for famous green tea.

Scholars, both domestically and internationally, have conducted extensive research on the application of deep learning in impurity detection. Liu et al. established a CPU Net semantic segmentation framework tailored for corn impurity assessment, incorporating a convolutional block attention mechanism and a pyramid pooling strategy within the U-Net architecture. The average MIoU, MPA, and ST of the model are 97.31%, 98.71%, and 158.4 ms, respectively, with a relative error of 4.64% compared to the manually calculated average [13]. Qi et al. developed a methodology for assessing wheat crushing rates and impurity levels utilizing the DeepLab-EDA semantic segmentation framework. This approach yielded a Mean Intersection over Union (MIoU) of 89.41%, a Mean Precision (MP) of 95.97%, and a Mean Recall (MR) of 94.83%, representing improvements of 9.94%, 7.41%, and 7.52% over the foundational model’s performance, respectively [14]. Rong et al. devised a dual-phase convolutional neural network architecture aimed at both segmenting images and conducting instantaneous detection of foreign materials within walnut visuals. Their technique demonstrates an impressive capability to accurately delineate 99.4% of the target areas in testing images while achieving a success rate of 96.5% in identifying contaminants during the validation phase [15]. Huang et al. proposed an impurity detection algorithm for Tieguanyin tea, building upon the foundation of the upgraded YOLOv5 model [16]. The enhanced model exhibits a greater confidence level than the baseline model in identifying tea contaminants, but the FPS is only 62.

The above research provides a reference for the research of this paper, however, the task of identifying impurities in prestigious green tea varieties differs significantly from the aforementioned research subjects and contexts, particularly given the challenges arising from impurities like tea stems, which closely resemble green tea in color and appearance, thereby significantly escalating the complexity of accurate detection. Currently, there are fewer research reports on deep learning in premium green tea impurity detection. In response to these hurdles, our research presents an enhanced YOLO model with model compression for detecting imperfections in high-quality green tea. The key contributions of this study are as follows: (1) To solve the two major problems of sample imbalance and scale sensitivity in tea impurity detection scenarios, four loss functions were compared experimentally, and Focaler_mpdiou was selected as the final loss function. The model achieved improved detection performance without increasing complexity. (2) To address the issue of high complexity and unsuitability for deployment in existing models, the L1 regularization pruning method is introduced to prune the model at the cost of sacrificing model detection accuracy and reducing model complexity. (3) In response to the dense and small target characteristics in tea impurity detection, the BCKD method is introduced to distill knowledge from the model, which improves the detection accuracy of small target impurities without increasing the complexity of the model.

## 2. Materials and Methods

### 2.1. Data Collection and Annotation

The tea used in this article is Rizhao green tea, sourced from the experimental base of the Tea Research Institute of the Shandong Academy of Agricultural Sciences (117° E, 36° N). The base is located in the southern part of the Shandong Peninsula and experiences a warm temperate humid monsoon climate with abundant sunshine and rainfall. In this experiment, four common impurities, including tea stems, tea fruits, melon seed shells, and stones, were evenly mixed into the tea sample. In April 2024, 1613 images of tea containing impurities were captured using a camera (Canon, EOS80D, Chengdu, China). During the collection process, the sample was placed on a white background with the camera perpendicular to the surface of the tea sample. The shooting distance was set to 30 cm, and the spatial distribution of tea leaves and impurities was randomly transformed. The samples were collected in two time periods: 9:00–11:00 in the morning and 14:00–16:00 in the afternoon, simulating subtle changes in lighting during different time periods to enhance the diversity of the image samples. A total of 1613 photographs were labeled using LabelImg software(1.8.1) to create a label file. The dataset was arbitrarily partitioned into training, validation, and testing subsets following a 6:2:2 distribution.

### 2.2. YOLOv8 Model

**Figure 1:**
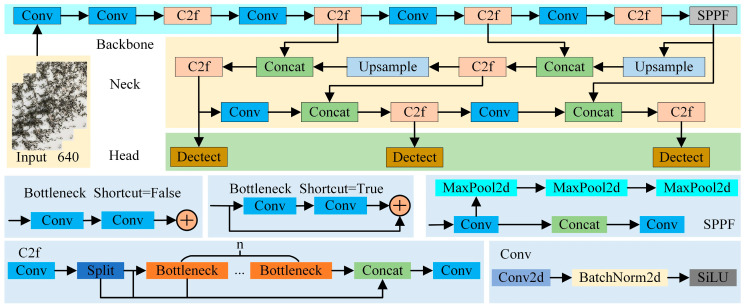
YOLOv8 structure diagram.

YOLOv8 builds upon the previous versions of the YOLO series, introducing innovative features and enhancements to boost performance and adaptability [17]. The architecture is depicted in Figure 1.

### 2.3. Focaler_mpdiou

To enhance border regression accuracy in varied detection tasks, the *IoU* loss is re-engineered through a linear interval mapping strategy, facilitating better focus on different regression samples [18]. The specific calculation is shown in Equation (1).

(1) $$\left\{\begin{array}{c} IoU^{Focaler}=\left\{\begin{array}{c} 0,\;\\ \frac{IoU-d}{u-d}\\ 1, \end{array}\right.,\begin{array}{c} IoU<d\\ d\ll IoU\ll u\\ IoU>u \end{array}\\ L_{Focaler-IoU}=1-{IoU}^{Focaler} \end{array}\right.$$

where $IoU^{Focaler}$ is Focaler-IoU after reconstruction, *IoU* is the initial *IoU*, and [*d*,*u*]ϵ[0,1], by modifying the parameters *d* and *u*, can allow $IoU^{Focaler}$ to concentrate on diverse regression instances.

There are two major problems with Focaler-IoU and MPDIoU [19] in tea impurity detection scenarios. First, the sample is imbalanced, with significant differences in the proportion of tea and impurities in the image, and border regression is susceptible to background interference. The second one is scale sensitivity, with a large range of impurity sizes, and traditional loss functions have insufficient accuracy for small target regression. To solve the above problems, the Focaler_mpdiou loss function is introduced. It applies Focaler-IoU to MPDIoU, and the specific calculation is shown in Equation (2).

(2) $$L_{Focaler\_mpdiou}=L_{MPDIoU}+IoU-{IoU}^{Focaler}$$

### 2.4. Model Compression

As deep learning advances at an accelerated pace, neural network architectures are becoming increasingly deeper, which cannot be easily deployed on resource-constrained devices. Therefore, a large number of model compression techniques have emerged, and model pruning and knowledge distillation are representative of them.

#### 2.4.1. Model Pruning

Deep learning network models often include a considerable number of superfluous parameters spanning from the convolutional layers to the fully connected layers, and many neuron activation values tend to cluster around zero. Model pruning, as one of the main ways of model lightweighting, reduces the model size by removing the unimportant connections in the network structure, and at the same time, it maintains the model’s accuracy at a level comparable to that of the original model. L1-regularized pruning reduces the parameter count in a model by setting the smaller weights in the model to zero. Its core idea is to prompt the model to learn sparse weight distributions by adding L1 regularization terms, which in turn enables pruning [20]. This article introduces the L1 pruning method, which reduces the complexity of the model while sacrificing the precision of the model measurement, achieving a lightweight effect of the model.

#### 2.4.2. Knowledge Distillation

**Figure 2:**
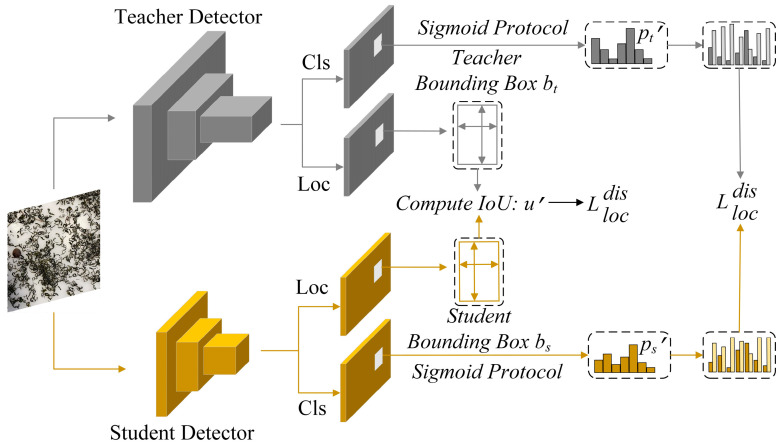
Schematic diagram of BCKD.

Knowledge distillation is a technique designed to transfer valuable information from a large-scale and intricate teacher model to a more compact student model, enabling the smaller model to mimic and leverage the capabilities of the larger one for comparable or superior performance while significantly reducing computational requirements. Bridging Cross-task Protocol Inconsistency for Distillation (BCKD) is a logical distillation approach presented at ICCV 2023 [21]. By incorporating binary classification distillation loss and IoU-based localization distillation loss, it effectively addresses the inapplicability problem of traditional knowledge distillation in dense target detection and significantly improves the performance and adaptability of target detection models. This method not only provides a new optimization direction in the field of target detection but also demonstrates the great potential of knowledge distillation technology in solving complex problems. The detailed procedure can be found in Figure 2. In response to the dense and small target characteristics in tea impurity detection, the BCKD knowledge distillation method is introduced to improve the detection accuracy of small target impurities without increasing model complexity.

## 3. Results and Analysis

### 3.1. Experimental Setup and Training Configurations

The experimental setup and training configurations employed in our research are detailed in Table 1.

### 3.2. Model Evaluation Metrics

Common target detection task evaluation indexes are used to assess the efficacy of the experimental outcomes. These performance assessments encompass precision (P), Recall (R), mAP, GFLOPs, Params, pruning rate, and FPS.

(1) P is determined by the ratio of true positive predictions (*TP*) to the total number of positive forecasts issued by the model (*TP + FP*).
(2) R is determined by the fraction of correctly identified positive cases (*TP*) out of all the actual positive instances in the dataset (*TP + FN*).
(3) mAP denotes the mean of all categories of detection accuracy and represents a composite measure of the average accuracy of detected targets.
(4) GFLOPs is a measure of the computational burden or intensity of the model.
(5) Params shows how complex and resource-intensive the model is.
(6) Pruning rate is the model’s pruning rate, illustrating the variation in computational requirements prior to and following model pruning.
(7) FPS is the refresh rate of the image.

The detailed computation method is presented in Equation (3).

(3) $$\left\{\begin{array}{c} Precision=\frac{TP}{TP+FP}\\ Recall=\frac{TP}{TP+FN}\\ mAP=\frac{1}{n}\sum_{i=1}^{n}AP_{i}\\ Pruning\;rate=\frac{GFLOPs\;prior\;to\;pruning}{GFLOPs\;following\;pruning} \end{array}\right.$$

where AP stands for the average precision of an individual category.

### 3.3. Evaluation Outcomes of YOLOv8 Models

YOLOv8 is classified into five models, namely, n, s, m, l, and x, based on the size of the model. Experiments were conducted on a homemade dataset containing impurity-laden tea for each of the five models, and the findings are tabulated in Table 2.

Upon examining and contrasting the empirical outcomes presented in Table 2, it becomes evident that as the model complexity grows larger, both the number of Params and GFLOPs increases, and the speed decreases. The P value of each model does not differ much, indicating that the models exhibit comparable accuracy in the detection of impurities. The R value for YOLOv8n is the lowest, while the R value for YOLOv8x is the highest, indicating that the YOLOv8x model can detect impurities with higher probability. The mAP value for YOLOv8n is the lowest, whereas that for YOLOv8s is the highest, and the YOLOv8s model achieves the greatest average accuracy for the detected targets. This study balances detection performance and model complexity. Although YOLOv8s achieves higher mAP, considering factors such as poor hardware performance in actual production, large models are difficult to deploy. Therefore, YOLOv8n has been selected as the foundational model.

### 3.4. Comparison of Experimental Results for Replacing the Loss Function 

The loss function was replaced for the selected base model, and the original loss function CIoU was replaced using ShapeIoU, SIoU, MPDIoU, and Focaler_mpdiou, respectively. The outcomes of these experiments are presented in Table 3.

Upon reviewing the experimental findings in Table 3, it becomes apparent that after substituting the loss function for the base model, there is a varying degree of enhancement in the model’s detection performance. It was only after substituting the loss function with Focaler_mpdiou that the model managed to enhance its performance. The model showed enhancements in P, R, mAP, and FPS while maintaining the same number of parameters and GFLOPs.

### 3.5. Teacher Model Selection

Focaler_mpdiou was used to replace the original loss function CIoU of other YOLOv8 models. The outcomes of these experiments are elaborated in Table 4.

As observed from the experimental results in Table 4, it is evident that after substituting the loss function for the remaining YOLOv8 models, only the P, R, and mAP of the YOLOv8m-Focaler_mpdiou model are better than the base model. So, the YOLOv8m-Focaler_mpdiou model was chosen as the teacher model.

### 3.6. Outcomes of the Model Pruning Experiments

The base model, after replacing the loss function, was pruned using the L1 regularization method at pruning rates of 1.4, 1.5, and 1.6. The findings from the experiments are presented in Table 5.

Upon examining the experimental results presented in Table 5, it is evident that after pruning the model at pruning rates of 1.4, 1.5, and 1.6 at the cost of a certain amount of detection accuracy, the model’s GFLOPs and Params are significantly reduced, resulting in an enhanced detection speed. In the three cases, the model with a pruning rate of 1.4 shows the optimal detection performance. So, this model was chosen as the student model.

**Figure 3:**
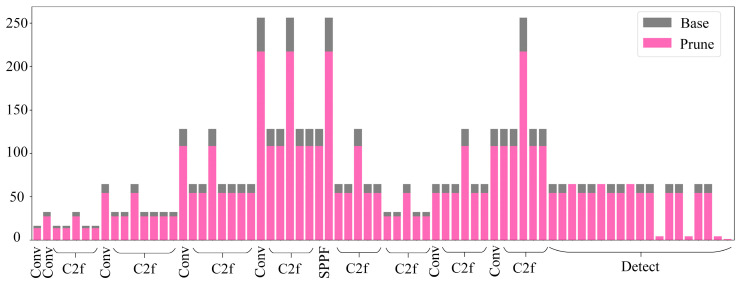
Changes in the channels prior to and following the pruning of the model.

Following the pruning of the model, Figure 3 depicts the alterations in the channel count across each layer. The gray color signifies the original number of channels per layer before pruning, whereas the pink color indicates the adjusted number of channels per layer post-pruning. An analysis of Figure 3 shows that after pruning the model, the number of channels decreases more, especially in the 8th layer of the model, the 21st layer of the C2f module, and the 9th layer of the SPPF module, indicating that these modules have a relatively small role in the model’s detection performance enhancement effect.

### 3.7. Results of Distillation Experiments

Knowledge distillation was carried out on the pruned model utilizing the BCKD approach, with a distillation loss ratio range of 0.1 to 2.0. The results of the experiments are displayed in Table 6.

After analyzing the experimental outcomes detailed in Table 6 above, it is evident that there was an enhancement in the model’s detection performance when distilling with the BCKD technique and the distillation loss ratio of 1.6 while maintaining the same number of Params and GFLOPs. Specifically, the P, R, mAP, and FPS of the model reached values of 0.9214, 0.8759, 0.9317, and 885.2, respectively.

### 3.8. Ablation Experiment

To validate the individual contributions of each altered component in this study, ablation experiments were conducted under the same conditions, and the findings are presented in Table 7.

Upon examining the experimental data provided in Table 7, it becomes clear that substituting the loss function can significantly enhance the model’s detection capabilities. This improvement is reflected in increases of 0.0019 in P, 0.0050 in R, 0.0070 in mAP, and 2.7 in FPS, without any increase in GFLOPs or the number of Params. Following model pruning, while there was a minor decline in detection performance, there was a substantial reduction in both GFLOPs and Params by 2.3 and 860350 B, respectively. Interestingly, R improved by 0.0029 despite pruning. A subsequent application of knowledge distillation further boosted detection performance without escalating GFLOPs or Params, resulting in enhancements of 0.0066 in P, 0.0041 in R, 0.0041 in mAP, and 5.3 in FPS. These findings confirm the efficacy of each individual modification in improving overall model performance.

### 3.9. Comparison Experiment

**Figure 4:**
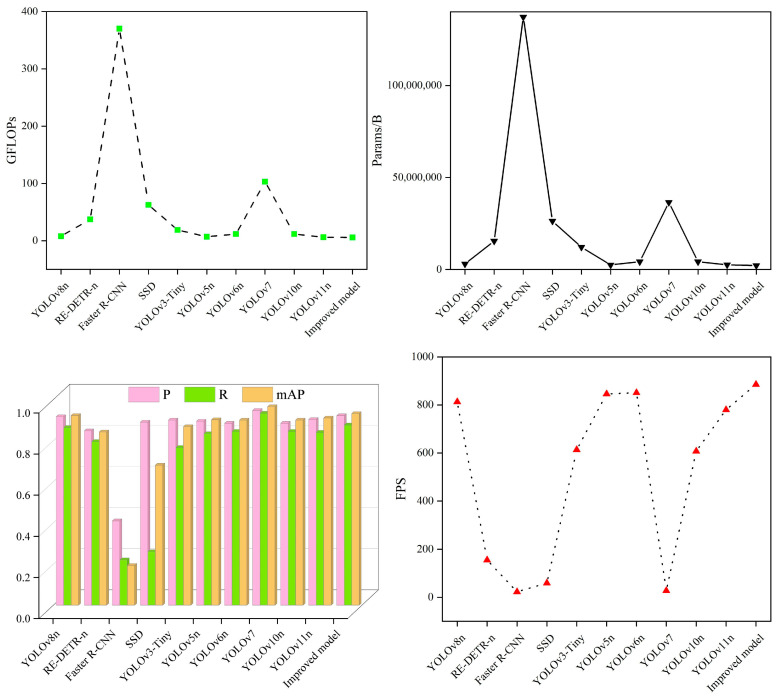
Comparative experimental diagram.

To more thoroughly assess the efficacy of the model introduced in this study, we compared it to mainstream target detection algorithms such as RE-DETR-n, Faster R-CNN, SSD, YOLOv3-Tiny, YOLOv5n, YOLOv6n, YOLOv7, YOLOv10n, and YOLOv11n. The experiments were carried out under identical conditions. The outcomes of these comparative experiments are summarized in Table 8 and Figure 4.

After examining the data in Table 8 and Figure 4, it becomes clear that compared to the improved models, the RE-DETR-n, Faster R-CNN, SSD, YOLOv3-Tiny, and YOLOv10n models have larger GFLOPs and Params, lower P, R, and mAP, and slower speed. The YOLOv5n, YOLOv6n, and YOLOv11n models demonstrate low detection accuracy, large model complexity, and comparable detection speed. Although the YOLOv7 model achieves high detection accuracy, it has a larger GFLOPs and a higher parameter count, which are 17.79 times and 17.01 times larger than those of the enhanced model, respectively. Additionally, its speed of detection is slower. The high-capacity architecture of YOLOv7 has generalization advantages in open scenarios, but there is a drawback of low efficiency in tea impurity detection. This study focuses on scene-specific feature extraction and lightweight deployment, and the model achieved more cost-effective performance in practical applications. In summary, the improved model also highlights certain advantages when compared to current mainstream models.

### 3.10. Comparison of Model Performance Prior to and Following the Enhancements

#### 3.10.1. Comparison of Detection Performance of Four Types of Impurities

**Figure 5:**
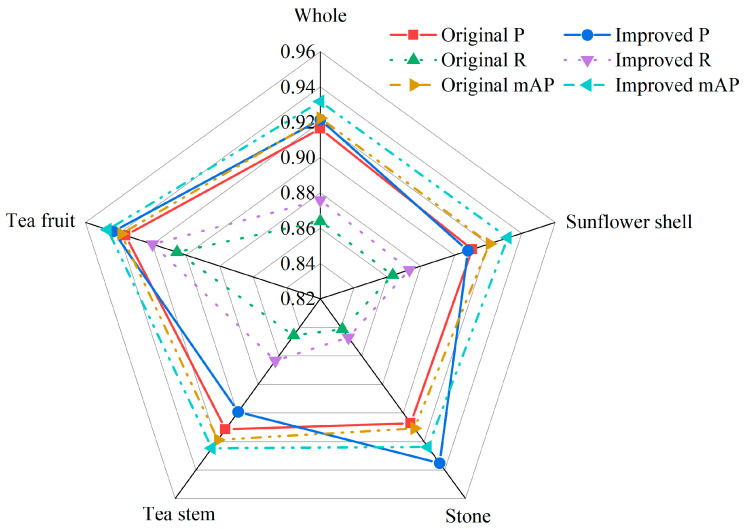
Comparison of detection performance.

The performance prior to and following the enhancement was visualized and compared, as illustrated in Figure 5. The improved model’s overall P value for the impurity detection of famous green tea was improved, suggesting that the model’s overall accuracy for impurity detection increased, but the P values of sunflower shells and tea stems were slightly reduced. The reason may be that the sunflower shells and tea stems are similar in color to famous green tea, and the improved model is not capable of learning their characteristics. The improved model showed greater detection of impurities based on its R and mAP values, indicating that the improved model can reduce the occurrence of the phenomenon of missed detection and improve the average detection accuracy.

#### 3.10.2. Heat Map Visualization Comparison

**Figure 6:**
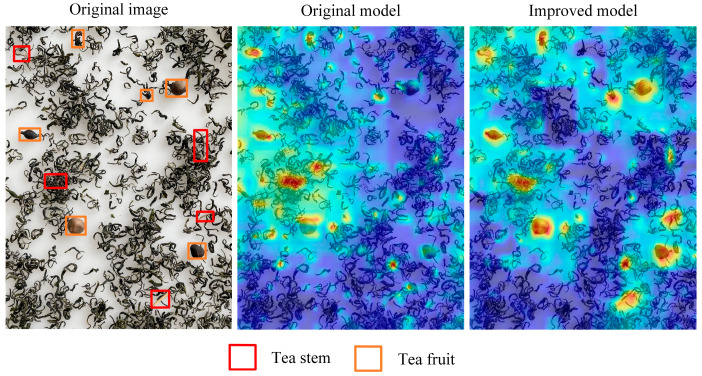
Comparison of the visualization of heat maps before and after improvement.

To examine how the model’s attention is distributed across an image, a test set image was selected at random to visualize its heat map, as illustrated in Figure 6. In the heat map, the depth of the color represents the network’s attention to the region, and a darker color indicates that the network focuses more on those areas. As shown in Figure 6, the improved model demonstrates greater attention to the impurity regions in famous green tea. This indicates that the improved model offers more thorough and precise detection of impurities.

#### 3.10.3. Comparison of the Model Detection Effect

**Figure 7:**
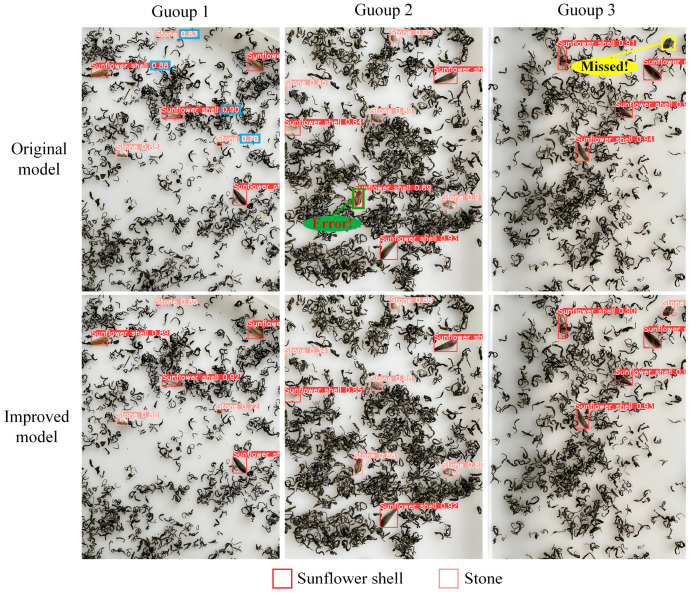
Comparison of model detection effectiveness prior to and following the enhancements.

For the purpose of evaluating the models’ detection capabilities both prior to and following enhancements, three images from the test set were randomly selected for comparison. As illustrated in Figure 7, the original model detection confidence is low in the first set, whereas the improved model detection confidence is improved in all of them. In the second scenario, the initial model has misdetection, while the enhanced model successfully avoids misdetection. In the third set, the original model has leakage detection, and the improved model effectively avoids leakage detection. The above results indicate that by enhancing the model, we not only increase the detection confidence but also reduce the cases of misdetection and omission.

### 3.11. Model Generalization Validation

**Figure 8:**
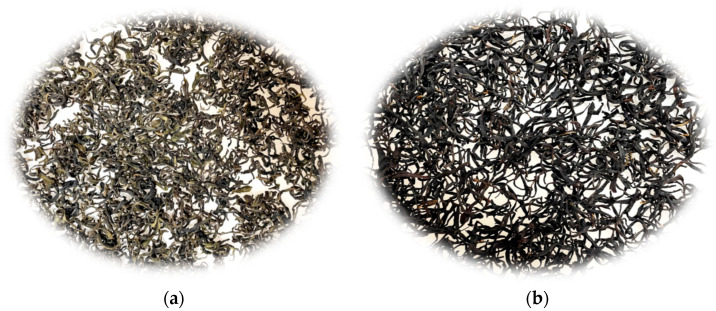
Samples of famous green tea and black tea. (**a**) Premium green tea, (**b**) Black tea.

Model generalization validation is a crucial step in the deep learning process. It aims to ensure that the constructed model not only performs well on training data but also has good predictive ability for unseen data. In this study, to verify the generalization ability of the impurity detection model for premium green tea, we conducted experiments on black tea samples, as shown in Figure 8, which depicts both premium green tea and black tea samples. From Figure 8, it can be seen that famous green tea (a) presents the characteristics of green, curly, and not tightly tied, while the black tea (b) presents the characteristics of black, curly, and tightly tied.

**Figure 9:**
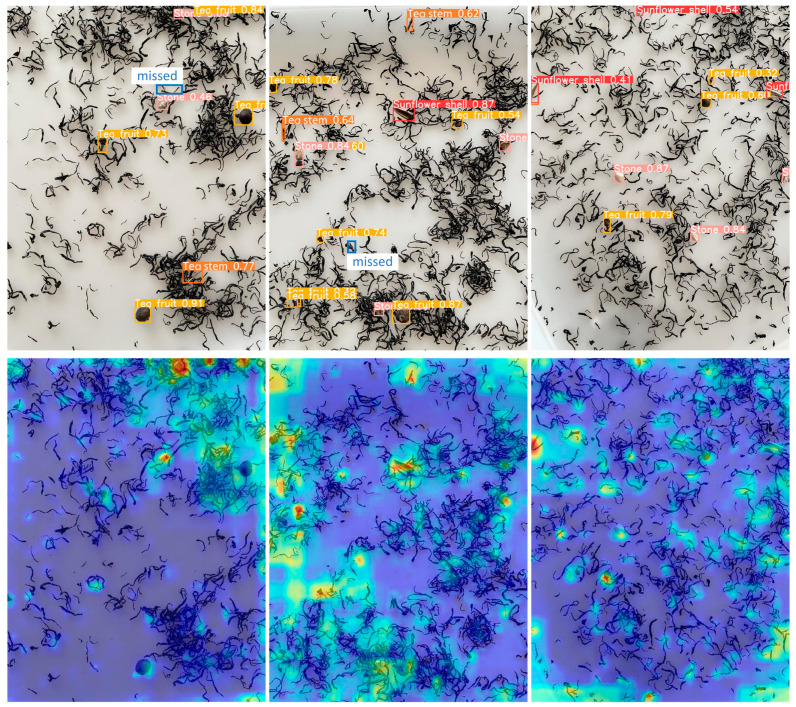
Model generalization validation effect.

The specific verification effect is shown in Figure 9. From Figure 9, it can be seen that the model missed the detection of tea stems, which may be due to the similar morphology of tea stems and black tea. Overall, the detection results of the model can correctly reflect the actual situation of impurities in black tea. This indicates that the model can adapt to the image features of black tea to a certain extent and effectively detect impurities, further demonstrating the model’s generalization ability in different tea categories. This provides a strong basis and practical reference for further optimizing the model and applying it to actual tea impurity detection.

## 4. Discussion

To solve the technical problem of high labor intensity and low sorting efficiency in manually sorting impurities in high-quality green tea, this study proposes an enhanced YOLO model with model compression for impurity detection in high-quality green tea. In comparison to the initial model, P, R, mAP, and FPS were enhanced by 0.0051, 0.012, 0.0094, and 72.2, while GFLOPs and Params were reduced by 2.3 and 860350 B, respectively. The model enhances detection precision while concurrently decreasing complexity, which fulfills the criteria for detecting and categorizing impurities in famous green tea. The overall P value of impurity identification in high-quality green tea was enhanced by improving the model, and overall, the accuracy of impurity detection was enhanced. From each type, the P value of sunflower shells and tea stems slightly decreased, which may be due to the fact that the color of sunflower shells and tea stems resembles that of high-quality green tea and that the improved model’s feature learning is not good enough. The enhanced model increased the R and mAP values for impurity identification in high-quality green tea, indicating that the enhanced model can reduce the occurrence of missed detections and improve the average detection accuracy. In the generalization experiment, the model may have missed the detection of tea stems, which may be due to the similar morphology of tea stems and black tea. Overall, the detection results of the model can correctly reflect the actual situation of impurities in black tea. This indicates that the model can adapt to the image features of black tea to a certain extent and effectively detect impurities in it.

Compared to traditional manual sorting methods, the detection and sorting method based on deep learning greatly improves sorting efficiency. Compared to Huang et al.’s research [16], this study greatly improved the detection speed and made the model more lightweight. Compared to Guo et al.’s research, this study has a lower cost and stronger anti-interference ability [8]. The improved model, due to its high detection speed and lightweight characteristics, can be directly embedded into tea impurity removal pipelines. In addition, although the improved model uses green tea as the training sample, it can still adapt to the image features of black tea in the generalization experiment and effectively detect impurities, proving the feasibility of cross-category detection.

Of course, the model proposed in this article also has certain limitations. Compared to the categories of tea impurities in actual production, the impurities selected in this article have a certain representativeness, but the types are relatively small, and the background environment is limited to white, making it difficult to fully simulate the complex situations that may occur in actual production environments.

## 5. Conclusions

This study proposes an enhanced YOLO model with model compression for impurities in high-quality green tea detection. In the homemade dataset containing four categories of impurities, i.e., tea stems, sunflower shells, stones, and tea fruits, experiments with YOLOv8 models were used to compare model complexity and the experimental results, using the YOLOv8n model as the base model. We experimentally compared ShapeIoU, SIoU, MPDIoU, and Focaler_mpdiou and selected Focaler_mpdiou as the final loss function. The loss function was replaced by Focaler_mpdiou for the other models of YOLOv8, and YOLOv8m-Focaler_mpdiou was selected as the teacher model based on the model’s detection capabilities. The model was pruned to achieve a lightweight model with a reduction in detection accuracy, and the model was subjected to knowledge model distillation without escalating the complexity to further enhance detection performance. Finally, the experimental outcomes based on the homemade tea impurity-containing dataset demonstrate that the GFLOPs, Params, P, R, mAP, and FPS of the enhanced model were 5.8, 2146078 B, 0.9214, 0.8759, 0.9317, and 885.2, respectively. The model enhanced detection precision while concurrently decreasing complexity. Afterwards, we conducted model generalization validation on black tea samples. The results indicate that the model can detect four types of impurities in black tea, but there are still missed detections. This model has the potential for industrial application in specific green tea categories and common impurity detection scenarios.

Our proposed impurity identification model for high-quality green tea improves detection performance while achieving model lightweighting. It also has certain advantages compared to some mainstream models currently available. However, the impurity identification model of famous green tea in our research has some limitations (e.g., it contains few types of tea impurities), which constrains the model’s ability to generalize. Going forward, we plan to expand the dataset to enhance the diversity of tea impurity data, enabling the model to detect multiple types of tea impurities in complex scenarios.

## Figures and Tables

**Table 1:** Experimental environment and training parameter settings.

Environment	Parameter Settings	Training	Parameter Settings
Operating System	Windows 10	Optimizer	Adam
CPU	13th Gen Intel Core i7-13700F	Epoch	200
GPU	NVIDIA GeForce RTX4070	Batch size	8
Deep learning framework	PyTorch2.0.0	Patience	50
Language	Python3.9		

**Table 2:** Evaluation outcomes of YOLOv8 models.

Model	GFLOPs	Params/B	P	R	mAP	FPS
YOLOv8n	8.1	3006428	0.9163	0.8639	0.9223	813.0
YOLOv8s	28.4	11127132	0.9203	0.8871	0.9409	349.3
YOLOv8m	78.7	25842076	0.9136	0.8814	0.9386	150.3
YOLOv8l	164.8	43609692	0.9213	0.8870	0.9393	89.5
YOLOv8x	257.4	68127420	0.9087	0.8965	0.9382	54.6

**Table 3:** Comparison of experimental results for replacing the loss function.

Model	GFLOPs	Params/B	P	R	mAP	FPS
YOLOv8n	8.1	3006428	0.9163	0.8639	0.9223	813.0
YOLOv8n-ShapeIoU			0.9267	0.8626	0.9253	804.3
YOLOv8n-SIoU			0.9042	0.8680	0.9225	808.9
YOLOv8n-MPDIoU			0.9104	0.8660	0.9220	812.8
YOLOv8n-Focaler_mpdiou			0.9182	0.8689	0.9293	815.7

**Table 4:** Experimental results for replacing the loss function for other YOLOv8 models.

Model	GFLOPs	Params/B	P	R	mAP	FPS
YOLOv8n-Focaler_mpdiou	8.1	3006428	0.9182	0.8689	0.9293	815.7
YOLOv8s-Focaler_mpdiou	28.4	11127132	0.9164	0.8783	0.9349	347.8
YOLOv8m-Focaler_mpdiou	78.7	25842076	0.9237	0.8884	0.9394	150.2
YOLOv8l-Focaler_mpdiou	164.8	43609692	0.9127	0.8966	0.9408	91.9
YOLOv8x-Focaler_mpdiou	257.4	68127420	0.9157	0.8931	0.9381	57.7

**Table 5:** Model pruning results.

Pruning Rate	GFLOPs	Params/B	mAP	FPS
YOLOv8n-Focaler_mpdiou	8.1	3006428	0.9293	815.7
1.4	5.8	2146078	0.9276	879.9
1.5	5.4	1991174	0.9262	927.4
1.6	5.0	1864591	0.9250	921.9

**Table 6:** Results of the knowledge distillation experiment.

Distillation Loss Ratio	GFLOPs	Params/B	P	R	mAP	FPS
0.1	5.8	2146078	0.9298	0.8660	0.9302	876.5
0.2			0.9249	0.8689	0.9308	875.3
0.3			0.9125	0.8742	0.9295	870.1
0.4			0.9276	0.8654	0.9298	871.5
0.5			0.9181	0.8765	0.9298	874.9
0.6			0.9146	0.8746	0.9287	874.6
0.7			0.9212	0.8680	0.9322	871.7
0.8			0.9176	0.8728	0.9299	875.1
0.9			0.9153	0.8770	0.9315	876.9
1.0			0.9036	0.8783	0.9303	874.5
1.1			0.9122	0.8756	0.9303	873.3
1.2			0.9117	0.8612	0.9295	876.5
1.3			0.9119	0.8732	0.9279	876.4
1.4			0.9137	0.8677	0.9298	880.3
1.5			0.9124	0.8713	0.9291	883.6
1.6			0.9214	0.8759	0.9317	885.2
1.7			0.9164	0.8621	0.9268	885.9
1.8			0.9138	0.8821	0.9320	859.1
1.9			0.9157	0.8721	0.9312	861.1
2.0			0.9143	0.8747	0.9311	858.6

**Table 7:** Results of the ablation experiment.

Baseline	Focaler_mpdiou	L1	BCKD	GFLOPs	Params/B	P	R	mAP	FPS
YOLOv8n				8.1	3006428	0.9163	0.8639	0.9223	813.0
	√			8.1	3006428	0.9182	0.8689	0.9293	815.7
	√	√		5.8	2146078	0.9148	0.8718	0.9276	879.9
	√	√	√	5.8	2146078	0.9214	0.8759	0.9317	885.2

**Table 8:** Comparison of experimental results.

Model	GFLOPs	Params/B	P	R	mAP	FPS
YOLOv8n	8.1	3006428	0.9163	0.8639	0.9223	813.0
RE-DETR-n	37.5	15492984	0.8473	0.7958	0.8421	155.5
Faster R-CNN	370.2	137098724	0.4091	0.2203	0.1934	23.1
SSD	62.7	26285486	0.8900	0.2604	0.6806	59.5
YOLOv3-Tiny	18.9	12129720	0.8999	0.7671	0.8676	613.3
YOLOv5n	7.1	2503724	0.8945	0.8339	0.9016	845.6
YOLOv6n	11.8	4234140	0.8846	0.8453	0.8999	850.9
YOLOv7	103.2	36497954	0.9470	0.9333	0.9653	27.8
YOLOv10n	11.8	4234140	0.8846	0.8453	0.8999	607.4
YOLOv11n	6.3	2582932	0.9029	0.8403	0.9104	779.5
Improved model	5.8	2146078	0.9214	0.8759	0.9317	885.2

## Data Availability

The original contributions presented in the study are included in the article, further inquiries can be directed to the corresponding authors.
